# The role of tumor microenvironment in drug resistance: emerging technologies to unravel breast cancer heterogeneity

**DOI:** 10.3389/fonc.2023.1170264

**Published:** 2023-05-17

**Authors:** Vincenzo Salemme, Giorgia Centonze, Lidia Avalle, Dora Natalini, Alessio Piccolantonio, Pietro Arina, Alessandro Morellato, Ugo Ala, Daniela Taverna, Emilia Turco, Paola Defilippi

**Affiliations:** ^1^ Department of Molecular Biotechnology and Health Sciences, University of Turin, Turin, Italy; ^2^ Molecular Biotechnology Center (MBC) “Guido Tarone”, Turin, Italy; ^3^ UCL, Bloomsbury Institute of Intensive Care Medicine, Division of Medicine, University College London, London, United Kingdom; ^4^ Department of Veterinary Sciences, University of Turin, Grugliasco, TO, Italy

**Keywords:** tumor microenvironment, cancer heterogenicity, drug resistance, molecular mechanisms, breast cancer

## Abstract

Breast cancer is a highly heterogeneous disease, at both inter- and intra-tumor levels, and this heterogeneity is a crucial determinant of malignant progression and response to treatments. In addition to genetic diversity and plasticity of cancer cells, the tumor microenvironment contributes to tumor heterogeneity shaping the physical and biological surroundings of the tumor. The activity of certain types of immune, endothelial or mesenchymal cells in the microenvironment can change the effectiveness of cancer therapies via a plethora of different mechanisms. Therefore, deciphering the interactions between the distinct cell types, their spatial organization and their specific contribution to tumor growth and drug sensitivity is still a major challenge. Dissecting intra-tumor heterogeneity is currently an urgent need to better define breast cancer biology and to develop therapeutic strategies targeting the microenvironment as helpful tools for combined and personalized treatment. In this review, we analyze the mechanisms by which the tumor microenvironment affects the characteristics of tumor heterogeneity that ultimately result in drug resistance, and we outline state of the art preclinical models and emerging technologies that will be instrumental in unraveling the impact of the tumor microenvironment on resistance to therapies.

## 1 Introduction

Breast cancer (BC) is the second leading cause of cancer death in women. Data from WHO (World Health Organization) reported about 2.3 million new cases and about 685,000 deaths from BC globally (1). Similarly, American Cancer Society’s projections for BC incidence in the United States in 2023 (https://www.cancer.org/cancer/breast-cancer/about/how-common-is-breast-cancer.html), estimate about 297,790 new cases of invasive BC in women, about 55,720 new diagnosis of ductal carcinoma *in situ* (DCIS), and about 43,700 death from this disease. The same statistics indicate for 2023 more than 3.8 million BC survivors in the United States, and 7.8 million worldwide, including both patients currently being treated and making this type of cancer the most prevalent worldwide. The median age at the time of diagnosis is 62 years and a woman’s lifetime risk of acquiring breast cancer in the United States is around 13%, with incidence rates rising by 0.5% annually in recent years. Currently, a woman’s chance of dying from BC is around 2.5%, death rates have been decreased due to improved therapeutic regimens, as well as earlier BC detection through screening programs and increased awareness. However, in recent years, the trend has marginally halted.

The breast cancer mass is composed not only by epithelial cancer cells, but also by a plethora of heterogeneous populations coming from the host, including endothelial cells, stromal fibroblasts, and a variety of immune cells that form the so-called tumor microenvironment (TME) (2, 3). The TME is a highly complex biological community embedded in a composite matrix of structural proteins constituting the extracellular matrix (ECM), in which immune cells (including macrophages, polymorphonuclear cells, mast cells, natural killer cells, dendritic cells (DCs), and T and B lymphocytes) and non-immune cells (such as endothelial cells and stromal cells) establish subtle interactions with cancer cells. This cellular cross-talk is based on the production of specific soluble (growth factors and cytokines) and insoluble (ECM proteins) molecules, and it determines the tumor’s natural history.

BC comprises numerous subtypes that differ genetically, pathologically, and clinically. Indeed, it is currently considered a group of neoplasms originating from mammary gland epithelial cells caused by a variety of genetic alterations, with different disease courses, responses to treatments, and clinical outcomes. This was best exemplified by next-generation sequencing studies depicting comprehensive molecular BC portraits in Cancer Genome Atlas (4, 5) and identifying more than 1600 likely driver mutations in 93 BC genes (6). BC can have distinct molecular profiles from one another, leading to a complex heterogeneity of tumor cell subpopulations within single tumors, between primary tumors and their metastasis, or between independent metastasis, as a consequence of tumor clonal evolution (7, 8). In addition to clonal evolution, tumor heterogeneity can occur also at the level of cancer cell plasticity. The capability of BC cells to reprogram their gene expression and change their behavior when triggered by internal or external stimuli coming from surrounding cells and secreted factors, provides dynamic and context-dependent features to tumor heterogeneity (9, 10). Moreover, heterogeneity is also modulated by the different composition of the TME, with different ratio between tumor-infiltrating lymphocytes, myeloid cells, macrophages (3), with the increased presence of cancer-associated fibroblasts (CAFs) (11) and endothelial cells that controls cancer cell properties. The heterogeneity in components of the BC mass can be either observed between the different BC subtypes, known as inter-tumor heterogeneity, or within the same tumors, known as intra-tumor heterogeneity (12).

Therapeutic approaches are still currently largely based on clinical and pathological BC features, mostly on the presence or absence of targets like the hormone receptors or the Human Epidermal growth factor Receptor 2 (HER2) (13), and they are not yet tailored to individual patients. In particular, endocrine therapy is expected for hormone-dependent BC patients, targeted therapy with monoclonal antibodies for HER2-positive patients, and chemotherapy for TNBC patients. However, the different mechanisms that contribute to the inter- and intra-tumor heterogeneity are responsible for tumor escape from therapeutic interventions.

Drug resistance is among the major obstacles to reach a long-term cure, and overcoming this problem is the biggest challenge in BC research today. Indeed, the heterogeneous pattern of molecular aberrations found in each cancer plays a crucial role in the resistance to anticancer treatment (14–16). The goal of cancer therapy is to target a population of cancer cells within a particular host environment. The pharmacological properties of the therapy, together with intrinsic and acquired molecular features of cancer cells, controlled also by the TME components, dictate the therapy’s efficacy. Unfortunately, despite the clinical management of BC improving every day, the number of patients developing drug-resistant tumors is still high (17). The resistance can be already present before the treatment (innate) or appear after the treatment administration (acquired) (18–20). The innate resistance is mainly due to intrinsic tumor heterogeneity: in primary cancer one or more subpopulations (e.g., Cancer Stem Cells) are resistant to the treatments from the beginning; on the contrary, the acquired resistance becomes evident after the therapy. In the clinical setting, innate and acquired resistance may coexist, making the long-term fight against cancer more complex.

In BC, standard chemotherapies and targeted therapies have both been extensively correlated to the escape of tumor cells that shape the clonal evolution of tumors, giving rise to drug-resistant subclones (21–23). Moreover, a comparison of the genetic diversity between pre- and post-treatment in tumor specimens indicates the role of therapy in selectively expanding resistant cancer clones that were initially present but at low frequency (14). In this context, TME cells play an important role in mediating the drug response and educating the cancer cells to become resistant to the therapy through extensive molecular crosstalk that we will discuss below (24–26).

We will first describe here what is currently known regarding inter-tumor and intra-tumor heterogeneity and the impact of TME on cancer progression and drug resistance. Moreover we will discuss the up-to-date tools for studying these complex interactions in preclinical models and in patient derived samples in cancer progression and drug resistance. We will present emerging technologies, such as the spatial location of tumor subclones and TME cells within their native spatial context. We will show how the rapid growth of these techniques together with the multi-omics conjoint analysis mode and deep learning network architecture, promise to provide a more comprehensive understanding of cell-to-cell variation within and between individual tumors.

## 2 Heterogeneity in breast cancer

### 2.1 Inter-tumor heterogeneity

Surgeons and pathologists have long reported BC heterogeneity, and its classification system has been continuously updated as knowledge of cancer cell biology increases (27–29). To be exploited as a prognostic factor (to estimate disease outcome of newly diagnosed patients) and predictive factor (to predict response to specific treatment), the classification system has been integrated with information on patient treatments and survival. Classical histopathologic evaluation distinguished preinvasive (*in situ*) and invasive BC based on their morphology and structural organization, classifying the vast majority of tumors as invasive ductal carcinoma not otherwise specified (IDC NOS, 75%), invasive lobular carcinoma (ILC, 15%) and other special subtypes of BC, rare and significantly different in terms of prognosis and response to treatment (30).

**Figure 1:**
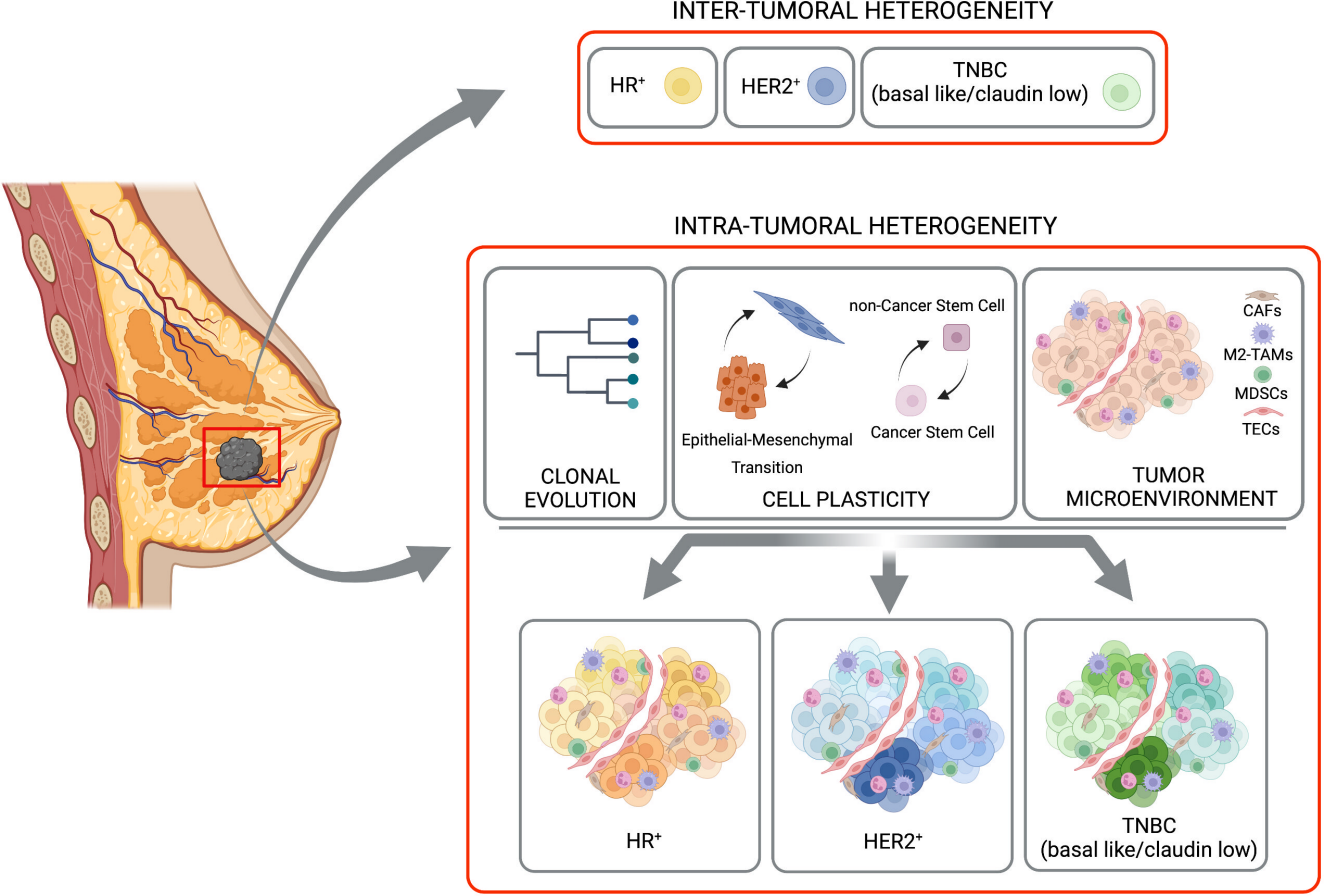
Inter-tumoral and intra-tumoral heterogeneity. BC is subdivided in three major subtypes based with inter-tumoral heterogeneity, on the expression of hormone receptor (HR-positive, HR+), HER2 receptor (HER2-positive, HER2+) or their complete absence (TNBC, basal like/claudin low). A strong heterogeneity inside each of these subtypes (the Intra-Tumoral Heterogeneity) is caused mainly by clonal evolution, cell plasticity (in terms of transition between Epithelial and Mesenchimal and/or Cancer Stem Cells and non-Cancer Stem Cells), and tumor microenvironment. Created with BioRender.com.

Immunohistopathologic classification, based on the expression/absence of Estrogen Receptor (ER), Progesterone Receptor (PR), or receptor tyrosine kinase HER2, allowed the definition of the major BC subtypes (**Figure 1**). This classification has strong prognostic and predictive significance, and it is critical together with grade and stage in the selection of targeted therapeutic options for every patient (31). The expression of these biomarkers is highly variable between tumors, with ER/PR positive cells ranging from 1 to 100 percent, where a frequency of stained cells higher than 1% in tumor biopsy is considered a cutoff for ER/PR positivity. In addition, HER2 expression is heterogeneous, and its positivity is accompanied by a score that integrates the percentage of positive cells, staining intensity, and membrane distribution (31). The concomitant lack of ER, PR, and HER2 defines Triple- Negative Breast Cancer (TNBC), a subtype that comprises 15-20% of all BC, highly prevalent in women younger than 40, Black, or with *BRCA1* gene mutation, and represents the most challenging BC to be treated. Molecular characterization of BC, based on gene expression profiling (32) and the definition of distinct transcriptional signatures, provided intrinsic molecular subtypes that partially recapitulated the histological classification (33): 1. luminal A (ER-positive/PR-positive, enriched in genes regulated by ER signaling pathway), 2. luminal B (ER-positive/PR-negative, HER2-positive or negative, enriched in genes regulated by ER signaling pathway and proliferation-associated genes), 3. HER2 enriched (HER2-positive, HER2-related gene expression, ER and PR-positive, and ER and PR-negative), 4. Basal-like (enrichment for genes expressed in basal epithelial cells, 70% of them are TNBC), 5. Claudin low (stem-like and Epithelial-to-Mesenchymal Transition-like signatures, mainly TNBC) (34). Contributing to heterogeneity, several genes are mutated, amplified, or deleted in various subtypes of BC and can be considered as drivers, the top 10 most frequent being: *TP53, PIK3CA, MYC, CCND1, PTEN, ERBB2, ZNF703/FGFR1* locus, *GATA3, RB1* and *MAP3K1* (6). *BRCA1* and *BRCA2* germline or somatic inactivating mutations, as well as methylation of the *BRCA1* promoter, also represent driver mutations for BC, usually associated with many genomic rearrangements. These different transforming events can originate in different cells of the mammary gland, and the differentiation state of these cells-of-origin plays a role in the determination of the tumor phenotype (31, 35, 36). Moreover, alterations in the expression of BC key genes have been reported as associated with epigenetic changes in DNA methylation and histone modifications, providing a further source of heterogeneity (30).

The metastatic progression of BC reflects its heterogeneous nature, with metastases to regional lymph nodes and in different distant organs such as bone, liver, lung, and brain. Association of molecular BC subtypes and metastatic sites has been reported with an increased frequency of brain metastases in the basal-like subtype, while showing bone metastases in luminal A and B subtypes and soft tissue metastases in the ER-negative subtype. It is well accepted that metastases originate from subsets of cells within the primary lesion, and the “seed and soil” model suggests that metastasizing cells may find in different organs the local microenvironment (the so-called niche) that favors their growth, generating secondary lesions that are the results of complex context-dependent interactions (30, 37).

### 2.2 Intra-tumor heterogeneity

Beside the differences found between tumors in different patients, distinct tumor cell populations, with different molecular and phenotypic profiles have been clearly described within the same tumor specimen, adding a further level of complexity to BC biology. Here we will focus on clonal evolution and cell plasticity as sources of intra-tumor heterogeneity (**Figure 1**).

#### 2.2.1 Genetic diversity of cell subpopulations: clonal evolution

Tumor initiation and progression rely on stochastic mutational events that ultimately lead mutated cells to acquire advantageous properties in terms of cell proliferation, resistance to cell death, and resistance to therapy. Tumors are generally thought to originate from a single cell, in which genetic driver alterations are followed by the acquisition of genomic instability, generating spontaneous mutations that can confer competitive advantages and driving the evolution of subclones with different functional features (6, 38). As described in (8, 10, 39), tumor subclones can derive from the selective pressure of therapy and can acquire drug resistance through i) the selection of rare pre-existing subclones that are able to expand or ii) the presence of new genomic/transcriptomic/epigenetic aberrations contributing to the drug-resistant phenotype (40). However, the resistance can be pre-existing in a large majority of the cells, and therefore the therapy does not impact the frequency of subclones (10). Relapsed or metastatic BC largely share the vast majority of their genomic alterations with the corresponding primary disease indicating pre-existing resistant clones. However, many metastatic cancers also harbor additional mutations that were previously undetected or are subclonal in the primary disease (8, 41).

From clonal evolution emerges the concept of temporal heterogeneity that indicates that tumor composition constantly changes over time. Tumors are the result of constantly ongoing competition between subclones under the selective pressure exerted by other clones, TME interactions, and therapies (42–44). The spatial distribution of the different clones is an additional source of heterogeneity; indeed, multiple biopsies from primary BC showed a locally constrained expansion of subclones, implicating their clonal evolutionary outgrowth and suggesting that sampling of a particular tumor’s area can be misleading in its molecular characterization (9).

#### 2.2.2 Cell plasticity: cancer stem cells and EMT

The genetic information encoded in cells’ nuclei is far from representing the only determinant of their complex behaviors; indeed, regulation of gene expression in response to intrinsic signaling pathways and extracellular stimuli from the TME strongly dictate tumor cells’ phenotype.

For a long time, mammary tumors have been considered a hierarchical model, with some rare cells capable of self-renewing, the Cancer Stem Cells (CSCs), relatively quiescent and resistant to treatments at the top of the hierarchy, and a vast majority rapidly dividing non-CSCs (45). According to this model, only CSCs, due to their intrinsic properties, can give origin to new tumors, including metastasis and relapse, and by asymmetric division to all the heterogeneous cell types found in a tumor. These non-CSCs are rapidly proliferating but poorly tumorigenic, incapable of self-renewal, and intended to differentiate (46). As in many other cancer types, lineage tracing experiments revealed cell plasticity in BC, showing that in the mouse mammary tumor virus-polyoma middle tumor-antigen (MMTV-PyMT) mouse model of mammary tumor (see below), some CSCs can disappear and new CSCs can form, demonstrating that stem cell state is plastic and cells can dynamically transit between CSCs and non-CSCs (47). Tumor cells of different phenotypic states coexist and evolve within the same tumor leading to cell subpopulations with different functional properties. Indeed, cell subpopulations showing stem cell-, basal- or luminal-like features isolated from BC cell lines are capable of generating functionally competent cells of all three phenotypes in a stochastic manner. Interestingly, under specific environmental stimuli, all three subpopulations efficiently seeded tumors in xenografts models, showing the tumorigenic phenotype classically ascribed to CSCs (48). In BC, a CSC-like phenotype can be acquired by cancer cells upon the activation of the so-called Epithelial-to-Mesenchymal Transition (EMT), a transient developmental program that leads to the de-differentiation of tumor cells with the acquisition of mesenchymal features. During EMT, cell-cell contacts between epithelial cells are lost, and cancer cells acquire a migratory and invasive phenotype, which can be reverted to more epithelial states via Mesenchymal-to-Epithelial Transition (MET). EMT is emerging as a heterogeneous range of differentiation states rather than a binary process; indeed, distinct intermediate states have been described in BC, with similar tumor-initiating capabilities but different plasticity and invasive potential (49, 50). The plasticity described between various differentiation states is not exclusively intrinsic to cancer cells but is also sustained by signaling from the TME surrounding CSCs and is defined as the CSC niche. Interestingly, CSC themselves can reprogram stromal cells to further sustain their activity, not only in the primary tumors but also in distant organs, eventually priming them for metastatic colonization. Indeed, it has been shown that BC circulating cells can reach nearly every organ, but tissue-specific microenvironments play a differential role in their engraftment and generation of metastases (51).

## 3 Cellular components of the tumor microenvironment

**Figure 2:**
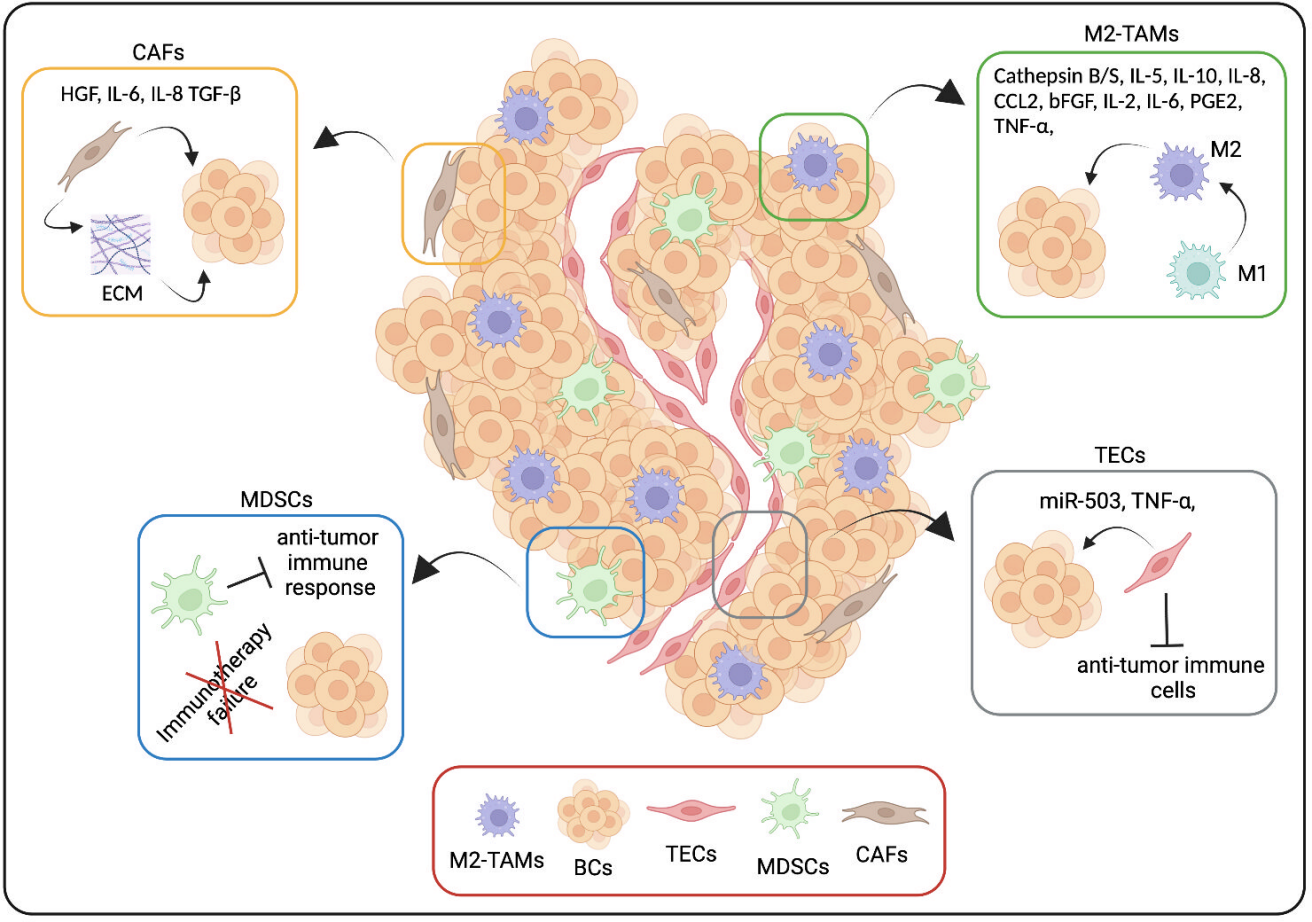
TME cellular components and drug resistance. We summarize here some molecular mechanisms through which TME cells, in particular Type 2 Tumor-Associated Macrophages (M2-TAMs), Myeloid-Derived Suppressor Cells (MDSCs), Cancer-associated Fibroblasts (CAFs) and Tumor Endothelial Cells (TECs) are able to induce drug resistance. Created with BioRender.com.

We will briefly introduce the main cellular components of TME cells, such as Type 2 Tumor-Associated Macrophages (M2-TAMs), Myeloid-Derived Suppressor Cells (MDSCs), Cancer-associated Fibroblasts (CAFs) and Tumor Endothelial Cells (TECs) ((**Figures 1**, **2**) (3, 11, 52–54).

### 3.1 Type 2-tumor associated macrophages M2-TAM

Tumor Associated Macrophages (TAMs) are an important and abundant immune component in the BC microenvironment. They mainly derive from circulating monocytes that reach the primary site, influencing several aspects of the tumor progression (53). Generally, TAMs have been classified as M1, with anti-tumor activity, or M2, with tumor sustaining roles.

### 3.2 Myeloid-derived suppressor cells

MDSCs are immature heterogeneous cells belonging to the myeloid family. Generally, they are subdivided into two main groups: polymorphonuclear (PMN) and monocytic (Mo) MDSC. The first population is characterized by the CD11b^+^Ly-6G^+^Ly-6C^low^ phenotype and the expression of high levels of arginase-1 (Arg-1). The second one is identified by the expression of CD11b^+^Ly-6G^low^Ly-6C^hi^ surface markers (55). As underlined by their name, the main feature of MDSCs is immunosuppression. In cancer, several soluble molecules, such as such as for example granulocytic-colony stimulating factor (G-CSF), C-X-C chemokine ligand (CXCL)2, CC-chemokine ligand (CCL)2, CCL5, CXCL5, and CXCL12 secreted by the tumor cause the block of myeloid differentiation, affecting their mobilization from the bone marrow and infiltration into the primary and secondary tumors (3). G-CSFAs underlined by their name, the main feature of MDSCs is immunosuppression. We recently described the ability of the adaptor protein p140Cap to counteract the mobilization and intratumor accumulation of polymorphonuclear myeloid-derived suppressive cells (PMN-MDSC), to prevent the establishment of a tumor conducive immune environment (56). (Salemme et al., 2023 in press).

### 3.3 Cancer-associated fibroblasts

Within BC TME, CAFs are a highly abundant and heterogeneous cell population belonging to the mesenchymal lineage. CAFs actively contribute to cancer progression via the production and remodeling of extracellular matrix components, secreted factors, and exosomes, influencing tumor growth and progression, angiogenesis, immune responses, and drug resistance (57) both in primary and metastatic lesions. Several hypotheses co-exist regarding CAFs’ origin, ranging from recruitment of bone marrow or adipose tissue-derived mesenchymal stem cells, EMT of tumor cells, activation of tissue-resident fibroblasts, to the trans-differentiation of endothelial cells. CAFs are also heterogeneous from the functional point of view, with a plethora of evidence showing their pro-tumorigenic effects (58) and some suggesting their tumor-constraining role in the early phases of tumorigenesis (59, 60). Limitation to our understanding of CAFs’ biology in BC derives from the lack of specific surface markers to identify and functionally characterize this heterogeneous cell type. Morphology is still the most consistent manner to distinguish CAFs within the TME, as commonly used biomarkers, such as α-smooth muscle actin (SMA), fibroblast-specific protein 1 (FSP-1/S100A4), or fibroblast activation protein (FAP), are neither all-encompassing nor completely specific, suggesting that CAFs include several subtypes of cells.

### 3.4 Tumor endothelial cells

The endothelium is a key component of the TME. Endothelial cells (ECs) play a role in regulating the exchanges between the bloodstream and the tissues. In pathological conditions, such as cancer, the TECs show a distinct phenotype at the molecular, structural, and functional levels. In particular, the vasculature becomes irregular, excessively fenestrated, and loose intercellular junctions, contributing in this way also to tumor growth, proliferation, dissemination, and metastasis (54).

## 4 TME mediated drug resistance

Overall, the cellular components of the TME engage in dynamic and extensive cross-talks based on both cell-cell interactions and paracrine signaling between each other and with the cancer cells, ultimately contributing to drug resistance with many mechanisms, some of which will be underlined below.

### 4.1 Type 2-tumor associated macrophages

TAMs protect cancer cells from drug attacks through the secretion into TME of numerous soluble factors, including enzymes, exosomes, interleukins, and chemokines. Shree et al. found that macrophages expressing cathepsin B and S protected BC cells against paclitaxel-induced cell death. Indeed, the combined administration of paclitaxel and cathepsin inhibitors can effectively enhance the therapeutic response (61).

Moreover, in BC, the treatment with cyclopamine, a known Hedgehog pathway inhibitor, increases the infiltration of M2-TAMs that, in turn, can limit the efficacy of chemotherapy by secreting Interleukin-6 (IL-6) (62). Interestingly, neutralizing antibodies directed against TAMs-derived Interleukin-10 (IL-10) significantly enhance the sensitivity of BC cells due to the reported relationship between the IL-10/STAT3/Bcl-2 signaling pathway and the BC cell resistance to paclitaxel treatment (63).

As a positive feedback loop between M2-TAMs and BC cells, the TAM-mediated secretion of the chemokine CCL2 contributes to the activation of the PI3K/Akt/mTOR pathway in BC cells, increasing their resistance to the anti-estrogen tamoxifen treatment. In contrast, tamoxifen-resistant BC cells secrete Tumor Necrosis Factor alpha (TNF-α), activate mTORC1-FOXK1, and promote TAMs M2 polarization that, in turn, secrete a high amount of CCL2 (64).

Another example of how TAMs are able to induce drug resistance is provided by Niu X. et al., reporting that the M2-TAMs activate the EGFR/PI3K/Akt pathway and, consequently, the sodium/glucose cotransporter 1 (SGLT1) expression to promote tamoxifen resistance in ER-positive BC cells (65).

Moreover, M2-TAMs, by secreting a variety of cytokines such as basic Fibroblast Growth Factor (bFGF), Interleukin-2 (IL-2), IL-6, TNF-α, prostaglandin 2 (PGE2) can trigger increased aromatase activity and estrogen production (66–68). TAMs could mediate doxorubicin and paclitaxel chemotherapy resistance through the secretion of high levels of IL-10 and activation of IL-10/IL-10 receptor/STAT3/Bcl-2 signaling pathway in TNBC cells (63, 69). In addition, in BC, resistance to carboplatin chemotherapy is related to M2-TAMs. Interestingly, in the study, the authors described that macrophages in the bone marrow stroma contribute to BC cell dormancy, leading to a CSC behavior. M2-TAMs and CSCs form intercellular gap junction communication, which is responsible for carboplatin resistance (70).

Several articles identify the M2-TAMs’ involvement also in resistance against targeted therapy. Ahmed S. et al. showed that, by secreting Interleukin-8 (IL-8), the TAMs activate Src/STAT3/ERK1/2-mediated EGFR signaling in BC cells, contributing to the resistance of HER2-positive BC to the small drug HER2 inhibitor lapatinib (71). Hu et al. described another interesting mechanism in which TAMs, after neoadjuvant treatment with the anti-HER2 humanized antibody trastuzumab, develop an immunosuppressive phenotype, upregulating B7-H4, a member of the B7 family of T cell costimulatory molecules, and causing the immune escape of HER2-positive BC cells (72). This TAMs’ “evolution” leads to a poor response after trastuzumab treatment.

The immunotherapy efficacy is also affected by the M2-TAMs infiltration into the primary tumor, as reported by Ekiz HA. et al. In particular, the expression of the receptor tyrosine kinase RON on macrophages inhibits the anti-tumor immune response enhancing the PDL-1 expression on TAMs as well as the Macrophage stimulating protein (MSP)-Macrophage Stimulating-1 Receptor (RON) signaling up-regulates the binding of CD80 and CTLA-4 to inhibit T cell activation, reducing the effectiveness of immune checkpoint inhibitors in the BC treatment (73).

Overall, the interactions between tumor cells and TAMs that promote TAMs to differentiate into immunosuppressive M2-polarized macrophages under treatment pressure play a role in drug resistance because M2-TAMs through the mechanisms above reported (and not only) are able to reduce the treatment efficacy.

### 4.2 Myeloid-derived suppressor cells

As underlined by their name, the main feature of MDSCs is immunosuppression; indeed, both PMN- and Mo-MDSCs are able to inhibit different types of immune cells, negatively impacting the ability of the host immune system to counteract the tumor progression and affecting the efficacy of the immunotherapy (3, 52, 74).

Thus, the MDSCs are the main obstacle to cancer immunotherapies, and the inhibition of their expansion/recruitment into the primary/secondary tumor sites may be a beneficial strategy for improving the efficiency of immunotherapeutic interventions.

### 4.3 Cancer-associated fibroblasts

In BC, different subsets of CAFs have been reported to accumulate differently in distinct subtypes and exhibit specific spatial distribution, with myofibroblastic subtypes accumulating in TNBC able to confer a tumor-suppressive TME (75). In particular, CAF secrete the C-X-C Motif Chemokine Ligand 12 CXCL12 attracting and retaining both myeloid (76) and CD4+/CD25+ T cells in the tumor, ultimately promoting their differentiation to Tregs immune cells and their survival via the expression of T cell interacting proteins (58, 75). BC CAFs have been characterized at the molecular level (77). Recent single-cell analysis of 768 CAFs isolated from the genetically engineered MMTV-PyMT preclinical model of BC reported three transcriptionally diverse subpopulations of CAFs, with a spatial separation of the CAF subclasses attributable to different origins, including the perivascular niche, the mammary fat pad, and the transformed epithelium. Notably, gene expression profiles of the three distinct CAFs classes correlate to different functional programs. Moreover, these profiles had independent prognostic values as biomarkers for metastatic disease and biomarker-driven development of drugs for precision targeting of CAFs.

The involvement of tumor stroma in BC prognosis is so evident that stromal gene expression can predict disease progression and clinical outcome independently of standard prognostic factors and published molecular signatures (11, 78–81). Moreover, in ER-negative BC, a stromal gene signature has been identified as associated with resistance to anthracycline-based neoadjuvant chemotherapy (82), with a predictive value for therapy response. CAFs’ role in conferring drug resistance has been observed in different tumors (83–86) and can occur via the release of paracrine survival factors or by activating pathways in tumor cells that ultimately lead to decreased chemosensitivity, such as the expansion of therapy-resistant tumor-initiating cells (87) and the enhanced expression of multidrug transporters (88). However, reflecting the heterogeneity of CAFs subpopulations and phenotypes, a few pieces of evidence indicating a role for tumor stroma in sensitizing BC to treatment have been reported (89).

A mechanism through which CAFs confer resistance to therapy is mediated by their role in the deposition and remodeling of extracellular matrix components. In particular, the integrins’ ligands collagens and fibronectin have been shown to be responsible for the decreased drug sensitivity of different BC cell lines to several treatments (i.e., paclitaxel, vincristine chemotherapy, tamoxifen, ionizing radiation, lapatinib, trastuzumab) through the activation of PI3K/AKT and Ras/Raf/MEK/ERK1-2 pathways (89). Tumor cells themselves can reprogram CAFs to increase the production of collagen, leading to the generation of a niche favoring the acquisition of CSC phenotype, resistance to chemotherapy (90), and driving tumor progression (91, 92). Moreover, CAFs release various soluble factors which contribute to the evasion of cancer cells from the cytotoxic effects of chemotherapy. CAF-secreted HGF and its receptor c-Met have been linked to increased resistance to EGFR and HER2 inhibitors in BC cells from different subtypes (89). An emerging technology based on microenvironment microarray (MEMA, consisting of printed ECM protein supplemented with soluble ligands) allowed to monitor of the growth of tyrosine-kinase inhibitors treated BC cells in more than 2500 different combinations of 56 soluble components and 46 matrix proteins of the TME (86). This study showed that specific soluble factors, highly expressed in BC CAFs, conferred lapatinib resistance to different BC cell types: in basal-like HER2-positive cells, HGF-mediated MET activation, while in luminal-like HER2-positive cells, neuregulin 1 beta (NRG1β), a ligand for the tyrosine kinase HER3, favored HER2-HER3 heterodimerization (86).

CAFs play a crucial role in sustaining tumor inflammation, engaging in intense cross-talk based on cytokines signaling with both TME components and tumor cells (11, 93, 94). As extensively reviewed by Dittmer and Leyh, cytokines such as the Tumor Growth Factor beta (TGF-β) and IL-6 are secreted by CAFs and contribute to drug resistance through both maintenances of CSCs and induction of EMT, whose key transcription factors (i.e., Snail, Twist) mediate the upregulation of transporters genes responsible for multidrug resistance. IL-6, IL-8, and complement cascade have been recently linked to CAF-mediated BC resistance to treatment. Indeed, a new subset of CAF defined as CD10+/GPR77+ (a C5a receptor) has been described as functionally relevant for stem cell maintenance. Niches formed by these CAFs foster the survival of CSCs, providing constant IL-6 and IL-8 secretion, which leads to persistent NF-kB signaling in BC cells and protects them from chemotherapy-induced cell death (88), regulating ABCG2 transporter expression.

A recent work exploited 3D co-cultures and microfluidic to unravel the dynamics of four TME cell populations (cancer, immune, endothelial cells, and fibroblasts) in the presence of the antibody-based HER2 targeting therapy Trastuzumab. Cell interactions have been visualized and quantified *ex vivo*, showing that Trastuzumab promotes longer interactions between cancer and immune cells that result in an anti-tumor ADCC (antibody-dependent cell-mediated cytotoxicity) immune response, while CAFs antagonized this effect (95) (see below). In line with this, recent evidence obtained analyzing 2 cohorts of Trastuzumab treated patients and a fully humanized immunocompetent *ex vivo* model of HER2-positive BC identified a population of TGF-β-activated CAFs specific of tumors resistant to Trastuzumab therapy (96). This CAF population has immunosuppressive functions associated with low IL-2 activity of functional relevance since antibody-based FAP-mediated stromal delivery of IL-2 in non-responsive tumors restored Trastuzumab efficacy (96). Another explanation for CAF-mediated Trastuzumab resistance resides in the newly identified subset of BC CAFs that express CD16 (also known as FcγRII, a cluster of differentiation molecule found on the surface of natural killer cells, neutrophils, monocytes, macrophages, and certain T cells) and which abundance in HER2-positive patients is associated with poor prognosis and resistance to Trastuzumab. The peculiar pro-tumor effect of CD16 in this CAF subpopulation has been explained by the ability of Trastuzumab-CD16 interaction to activate intracellular signaling involving the SYK-VAV2-RhoA-ROCK-MLC2-MRTF-A pathway, that ultimately leads to elevated contractile force, enhanced matrix production, and stiffness. Targeting of a Rho family guanine nucleotide exchange factor, VAV2, which is indispensable for the function of CD16 in fibroblasts rather than leukocytes, reverses desmoplasia provoked by CD16+ fibroblasts, revealing a role for the fibroblast FcγR in drug resistance, and suggesting that VAV2 is a promising target to enhance the effects of Trastuzumab treatment (97).

### 4.4 Tumor endothelial cells

Some evidence indicates an involvement of TECs in BC drug resistance. Bovy N. et al. reported that BC patients receiving neoadjuvant chemotherapy showed increased miR-503 production. Interestingly, the origin of this increased production is ascribed to the exosome released by TECs, mediating an acquired resistance (98). Moreover, the product of the NF-κB signaling cascade TNF-α was upregulated in BC ECs after doxorubicin chemotherapy treatment. In turn, TNF-α induces the overexpression of CXCL1/2 in BC cells that, through its receptor CXCCR-2, stimulates the CD11b+Gr1+ myeloid cells to secrete S100A8/9. The activation of the TNF-α-CXCL1/2-S100A8/9 paracrine network mediates the pro-survival effect in BC cells and drives chemoresistance by activating ERK1/2, p38 MAPK, and p70S6K (99). In addition, as an alternative pathway, TEC cells, through Notch signals, are able to promote BC stemness mediating the acquisition of resistance to therapy (100). An important role of TECs in drug resistance against immunotherapy has been described. In particular, TECs are able to favor the recruitment of immunosuppressive cells as well as to inhibit primary tumor infiltration by anti-tumor immune cells. The downregulation of the endothelial E-selectin/P-selectin, ICAM-1, and VCAM-1 proteins results in the inhibition of T cell adhesion as well as the altered chemokine expression such as the nitrosylation of CCL2 by reactive nitrogen species blocks CD8+ T cell recruitment while improving MDSCs recruitment. Moreover, the increased expression on TECs of both PD-L1/2 and FasL expression induces T cell exhaustion and apoptosis, respectively (101).

## 5 *In vivo* models to mimic BC complexity

**Figure 3:**
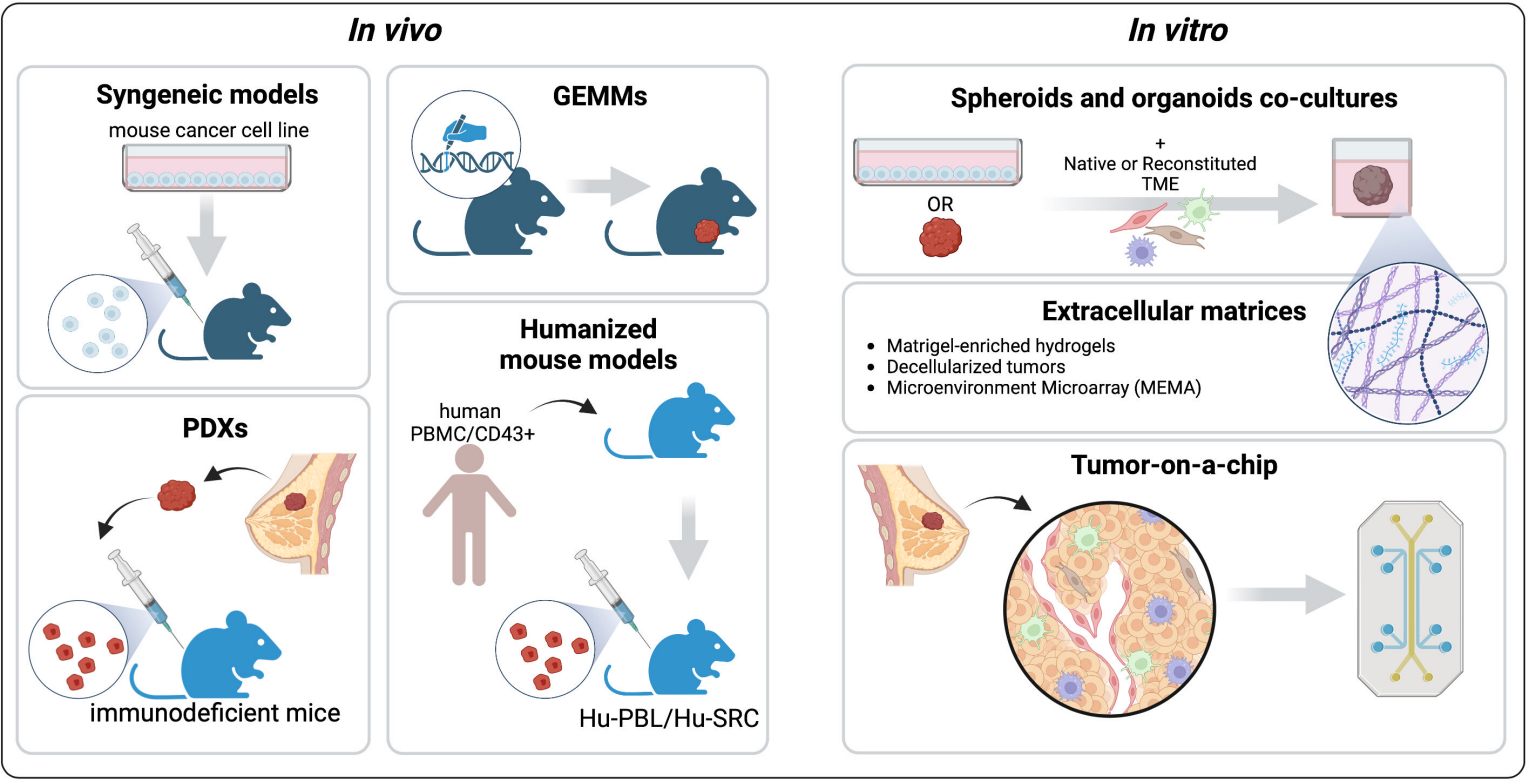
Pre-clinical models to recapitulate the TME. The common *in vivo* platforms that mimic different aspects of the BC TME are syngeneic Genetically Engineered Mouse Models (GEMMs), Patient-Derived Xenografts, and Humanized mouse models. Among the *in vitro* models we describe spheroids and organoids co-cultures with native or reconstituted TME components, engineered extracellular matrices (ECMs), and tumor-on-a-chip platforms. Created with BioRender.com.

Nowadays, preclinical mouse models are widely used to recapitulate the tumor complexity and how this complexity affects drug response. Accurately choosing the best model is crucial to translate the *in vivo* preclinical findings to patients (**Figure 3**).

### 5.1 Orthotopic injection in syngeneic mouse models

In syngeneic mouse models, mouse-derived BC cells are implanted orthotopically into immune-intact mice of the same genetic background. These immune-competent models allow the investigation of different aspects of the tumor-immune system crosstalk. The high engraftment rate and the rapid tumor growth make this model an attractive approach to studying BC biology and drug response. Nevertheless, a recent study by Zhong et al. revealed significant differences in the genomic, proteomic, and immunohistochemistry profile of a panel of ten commonly used syngeneic mouse models, including the most widely used BC syngeneic models EMT-6 and 4T1, compared to the subtype-matched human tumors (102). It is crucial to carefully consider that syngeneic mouse models of BC do not form these cancers spontaneously. Therefore, they do not recapitulate the complex interactions between tumor cells and the TME that characterize the slow evolution of human neoplasms (103). Indeed, most cell lines used for syngeneic BC models are derived from advanced tumors that have already undergone immune selection *in vivo*. Another aspect that should be thoughtfully evaluated when trying to recapitulate the human TME is optimizing the experimental design for the *in vivo* tumor growth of transplantable cell lines. In particular, injection of different numbers of cancer cells could detrimentally affect infiltrating leukocyte populations and response to immune checkpoint blockade (104).

### 5.2 Genetically-engineered mouse models

Genetically-engineered mouse models (GEMMs) develop cancer in an autochthonous manner upon overexpression of oncogenes or deletion of tumor suppressor genes (often combined) in a tissue-specific and temporally controlled manner (105). Compared to syngeneic models, GEMMs better recapitulate the multistep pathogenesis of BC and the crosstalk between neoplastic cells and the TME. Moreover, the competent immune system of GEMMs makes them uniquely suited for investigating cancer immunotherapy approaches. The Mammary-specific Polyomavirus Middle T antigen overexpression mouse model (MMTV-PyMT) is the most commonly used GEMM. Although the Middle T oncogene is not present in human tumors, its expression in the mammary epithelia induces transformation and generation of multifocal tumors that readily metastasize to the lungs without the need for additional mutations in metastasizing cells (42, 106). Interestingly, this murine model was used to demonstrate, via intercrossing with 27 different mouse strains, that the genetic background determines the age of tumor onset and the development of metastases, providing the first evidence that genetic heterogeneity plays an important role in tumor progression (42, 107).

According to a gene expression profile analysis, the PyMT tumor closely resembles the aggressive forms of the luminal B subtype of human BC, exhibiting loss of ER and PR expression and overexpression of HER2 and cyclin D1 with the progression of the disease (108). PyMT-derived BC tumors have provided significant genetic and mechanistic insights into breast tumorigenesis, as well as for preclinical testing of potential therapies (82). The K14cre BRCA1f/f Tp53f/f mouse model spontaneously develops tumors mimicking the human clinical features and genetics of basal-like/TNBC. This model has recently helped to provide new understandings into the crosstalk between cancer cells-intrinsic redox mechanisms and the formation of protumorigenic TME (110, 111). Specifically, activation of the transcription factor aryl hydrocarbon receptor by ROS promotes the production of chemokines to attract monocytes and activate the proangiogenic activity of macrophages (110).

### 5.3 Patient-derived and humanized mouse models

Preclinical mouse models that more likely recapitulate the intra-tumor and inter-tumor heterogeneity of human cancer are the patient-derived mouse models (PDXs). These models are becoming the standard platforms used for preclinical drug testing since they preserve the tumor architecture and the relative proportion of cancer cells and stromal cells. In PDXs, cancer tissue is implanted subcutaneously, orthotopically, or under kidney capsules in immunodeficient mice and can be serially transplanted. The ability to preserve the TMEs’ structure, the clonal genomic landscape, transcriptomic, epigenomic and signaling pathway signatures of the original parental tumors makes PDXs a valuable tool for precision medicine, enabling drug testing and resistance studies, assessment of tumor heterogeneity and evolution during disease progression (112–114). Indeed, they are currently used for co-clinical trials, whereby preclinical studies are conducted in parallel with human trials. Using an animal “avatar” allows the integration of valuable data in a real-time manner for each patient, thus enabling a more precise stratification and treatment customization. Many research centers and pharmaceutical companies have successfully developed and characterized PDXs as models for the different clinical and molecular subtypes of BC (115–119). More recently, an extensive collection of PDXs recapitulating the deadliest forms of BC has been generated (120). This platform includes drug-resistant, metastatic, endocrine-resistant estrogen ER+ and HER2-positive tumors, many of which are primary-metastatic pairs or longitudinal collections from an individual patient over time. Importantly, these PDXs reflect the intrinsic heterogeneity of the subtypes in terms of mutational signatures, paving the way for new therapeutic opportunities for these aggressive tumors (120). The significant disadvantage of PDXs in faithfully representing the TME is the lack of human immune system components, such as circulating T and B cells.

### 5.4 Humanized mouse models for cancer

Humanized mouse models have been developed to overcome species-specific differences in the genetics and immune system between mice and humans (121). Humanized mouse models of cancer are immunodeficient mice reconstituted with representative subsets of human immune cells and engrafted with human tumors (122). The engraftment of specific cell populations in mice will influence the relative abundance of different human immune cell types. Therefore, it is crucial to select the most appropriate humanized mouse platform to specifically address the experimental question and gain translational potential. Injection of human Peripheral Blood Mononuclear Cells (PBMCs) into immunodeficient SCID mice is the most straightforward method for developing humanized models, namely the Human Peripheral Blood Leukocyte (Hu-PBL) SCID mice. Hu-PBL-SCID mice are characterized by limited numbers of engrafted human myeloid cells and B cells. Conversely, CD4+ and CD8+ subsets of CD3+ T cells are abundant, and their expansion eventually develops an acute immune response against mouse MHC molecules, leading to xenogeneic Graft-Versus-Host Disease (GVHD) and restricting the experimental window for these animals to a few weeks (123). To overcome GVHD, genes encoding mouse MHC class I and II molecules have been inactivated, enabling a more extended time window for conducting experiments. Iizuka and coworkers took advantage of a human PBMC-transplanted MHC class I- and class II–deficient NOG mice engrafted with BC cell lines to test the cytotoxic activity of a bispecific antibody targeting human CD3 and B7-H4, considered to be a negative regulator of immune responses. B7-H4 is overexpressed in many human cancers, suggesting its potential role as a cancer therapy target. This therapeutic strategy resulted in enhanced tumor infiltration of activated CD8+ T cells and reduced tumor growth. Of note, because B7-H4 is highly expressed independently of HER2 or PD-L1 expression in breast cancers, they propose the use of this therapeutic agent for PD-L1−B7-H4-expressing tumors or anti-HER2 antibody nonresponsive breast tumors (124). An adequate immune reconstitution is achieved with the human Stem Repopulating Cell (Hu-SRC) mouse model, which results from the engraftment of immunodeficient mice with human CD34+ Hematopoietic Stem and Progenitor Cells (HSPCs). Hu-SRC engrafted with PDX or Cell line-Derived Xenografts (CDX) have been used to study the human immune system–tumor crosstalk, evaluate biomarkers, and the preclinical activity of immuno-oncology agents (125–127). For instance, Scherer and coworkers recently developed and characterized an immune-humanized PDX model of estrogen-independent endocrine-resistant ER-positive metastatic BC that harbors a naturally occurring ESR1 mutation (126). Mutant ESR1 promotes endocrine resistance since it renders the ER protein constitutively active and less dependent on estrogen for its function, limiting treatment options. Importantly, ESR1 mutant tumors gain basal-like features associated with increased immune activation, implicating potential immune therapeutic vulnerabilities that should be deeply investigated using immune humanized preclinical models (128). The limitations of this model are mainly the requirement of pre-experimental sub-lethal γ-irradiation to enable engraftment and the limited maturation of human T cells in the murine thymus (121). To promote the development of T cells in a human thymus-like environment, researchers have co-implanted human CD34+ HSPCs and autologous fetal thymus tissue into SCID mice, generating a mouse model named BLT (bone marrow - liver - thymus) (129).

## 6 *In vitro* models to mimic BC complexity

*In vitro* systems have a major advantage over mouse models: the ability to precisely control the experimental settings (**Figure 3**). Indeed, *in vitro* platforms can recapitulate different aspects of the TME, including the cellular compartments, physical properties, and chemical cues (130). Compared to cell lines grown in conventional 2D culture, 3D systems enable the integration of these elements, capturing more faithfully the intra-tumor heterogeneity and allowing the study of tumor-stromal interactions and drug responses. Here, the commonly used and emerging platforms to study the BC TME are presented (**Figure 3**).

### 6.1 Spheroids

To investigate the role of the TME in 3D conditions, researchers have developed a variety of protocols for the generation of spheroids, cellular entities cultured as free-floating aggregates, with or without the addition of extracellular matrix and growth factors (131). Mammospheres are BC spheroid cultures enriched in progenitor cells that differentiate along multiple lineages. To this concern, mammospheres derived from freshly isolated BC samples exhibit CSC-like properties and multiple drug resistances (132). L. Hamm and coworkers established a high throughput tumor spheroid microprinting technology to produce homogeneously-sized spheroids to model the interaction of CAFs and TNBC cells and examine drug resistance (133). In another work, the 3D bioprint technology was leveraged to manufacture a 3D structure containing BC cells in the core and adipose-derived mesenchymal stem/stromal cells (ADMSCs) in the edges. The authors proposed the use of this 3D model for chemoresistance studies (134).

### 6.2 Patient-derived organoids

Tumor organoids are complex 3D structures that originate from dissociated tumor tissues or circulating tumor cells that are embedded into the bio-mimetic matrices with growth factor supplements to encourage a self-organizing process (135). Compared to spheroids, they better resemble the original tissue both histologically and genetically Patient-derived organoids (PDOs) largely retain the parental tumor heterogeneity, therefore providing the enormous potential to understand resistance mechanisms and predict response to treatment in individual patients (136, 137). Organoids can be cryopreserved and expanded for long-term culture. Of note, banks of human BC organoids are currently available (120, 138). In particular, the collection of nine sets of matched human BC tumors, PDXs, and PDOs generated by Guillen and coworkers represent a promising platform for drug screening treatment-resistant tumors (120). Primary PDOs contain subsets of stromal cells, including fibroblast and immune cells; however, these cells are gradually lost during the long-term culture (139). Nevertheless, researchers have grown organoids with native or reconstituted TME elements (140). Recently, Rivas and coworkers developed an *ex vivo* 3D model of HER2-positive BC that recapitulates patients’ response to treatment, consisting of fluorescent human HER2-positive BC cells co-cultured with patient-derived fibroblasts and naïve primary immune cells collected from the peripheral blood of healthy donors (96).

### 6.3 Mimicking the ECM

Organoids are commonly cultured in hydrogels enriched in extracts of ECM proteins. Among these, the basement membrane extract Matrigel is considered the gold standard for supporting tumor organoids’ growth. However, Matrigel typically suffers from inherent compositional variation and lot-to-lot variability, which can confound analysis and affect the model’s reproducibility (141). As an alternative biomaterial, decellularized breast tumors or Patient-Derived Scaffolds (PDSs) have been used to better recapitulate the native tumor ecosystem. In recent work, BC PDSs were recellularized with cancer cell lines as platforms for drug testing, revealing that MCF7 cells enhanced their resistance against the conventional chemotherapy drugs 5‐fluorouracil, doxorubicin, and paclitaxel in comparison to 2D cultures (142). These data suggest that PDSs could be exploited to examine the effects of the ECM on cancer drug responses in the clinical setting and may represent a significant step forward in the field of personalized medicine (143). The MEMA platform is used to interrogate the impact of thousands of microenvironmental proteins on the phenotype of different cancer cells, including primary cells and cell lines (144). By printing specific and defined combinations of functional proteins into well plates, it was possible to study microenvironment effects on anti-HER2 tyrosine-kinase inhibitors response (86).

### 6.4 Tumor-on-a-chip

Recent advances in tissue engineering technology allow the development of organ-on-a-chip devices in which cancer cells are grown in a dynamic environment consisting of microchannels that can be perfused at tailorable flow rates. The ability to finely control mechanical stress, shear flow, and concentration gradients makes the organ-on-a-chip technology particularly useful for studying angiogenesis, metastasis, mechanotransduction pathways, and cancer cell behavior under shear stress (145). However, several on-chip models were designed to mimic tumor-stromal cell interactions. For example, Aung and coworkers, taking advantage of a BC-on-a-chip model consisting of a heterogeneous mix of cells and noncellular elements, investigated the role of tumor-associated hypoxia and the BC-immune cell interaction on T lymphocyte recruitment (146). BC-on-a-chip platforms could represent powerful *ex vivo* platforms to study, within immunocompetent settings, drug responses that depend on the TME (95). Moreover, it is possible to integrate these platforms with advanced live cell microscopy technologies and automated image analysis to capture the behavior of single cells in the tumor ecosystem and the cell-cell interactions (95).

## 7 Emerging technologies to study BC complexity

Tumor profiling is a powerful tool to dissect key molecular signatures of cancer cells and deeply investigate the sources of diseases. The role of large landmark projects, such as The Cancer Genome Atlas Program (TCGA (4)) and Molecular Taxonomy of Breast Cancer International Consortium (METABRIC (147)), and of specific analysis strategies, such as GSEA (148, 149), have allowed scientists to begin to approach the complexity of the tumor system in question, enabling accurate and precise stratification of patients. Subsequent meta-analyses based on separate BC and TME data showed that very different results emerge from the bulk transcriptomic data (150).

**Figure 4:**
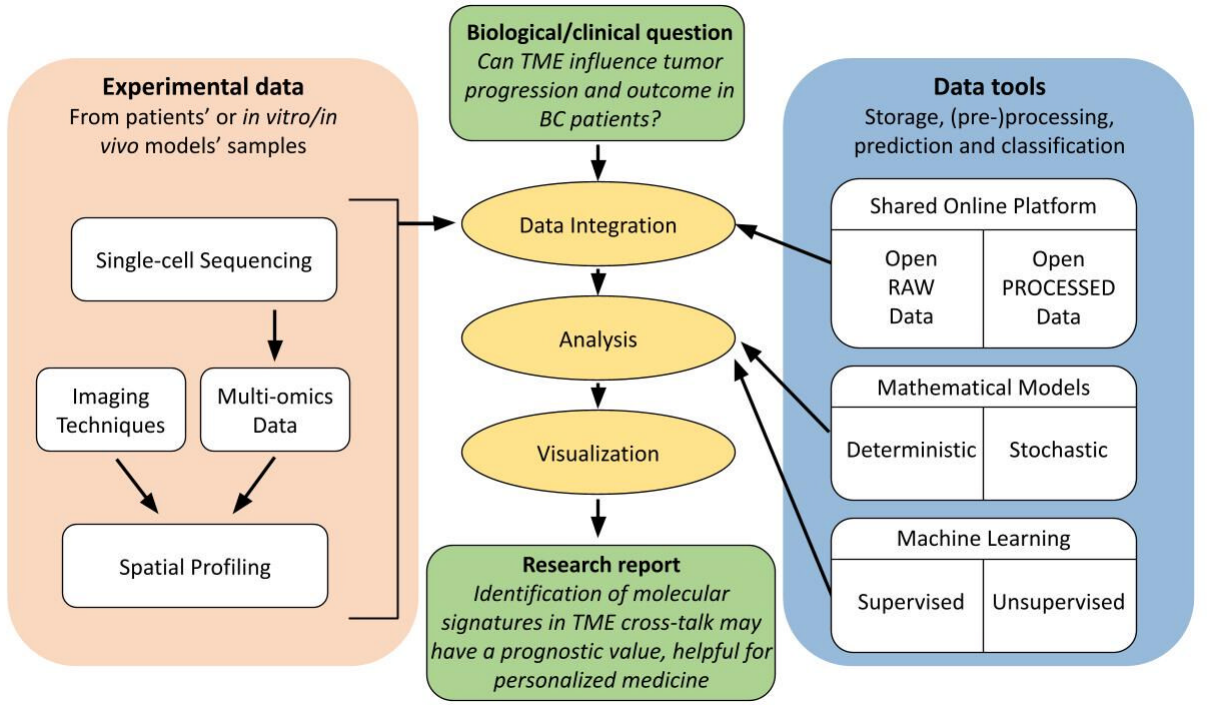
Flow diagram of good practice guidelines for spatial analysis users. Schematic representation of complexity and interconnectivity of single-cell multi-omics data in spatial analysis, from experimental data to scientific report to address a biological/clinical investigation. These techniques benefit from an integration with relative online available data and from an extensive computational analysis in order to increase the accuracy, reproducibility and reliability of the obtained results in a specific research field.

Single-cell analysis refers to the investigation of individual cells to obtain genomic, transcriptomic, or multi-omics information at the single-cell level. The data obtained with these technologies have a much higher resolution than conventional bulk sequencing methods in terms of the number of cells. Taking advantage of these emerging technologies, including spatial analysis and artificial intelligence, it is possible to identify comprehensive biomarkers allowing more precise patient stratification, signal resistance identification as it begins, and relapse prediction (151, 152) (**Figure 4**) A brief description of emerging technologies for unravelling breast cancer complexity is included in **Table 1**.

**Table 1:** Brief description of emerging technologies for unravelling breast cancer complexity.

Technologies	Description	Advantages	Limits	References
Single-cell sequencing	Genomic, transcriptomic, or other-omics information at the single-cell level.	•Higher resolution compared to conventional bulk sequencing methods in terms of the number of cells. •Heterogeneity dissected at single-cell resolution. •Analysis of gene expression changes upon drug. treatment. •Relatively low cost. •Can be integrated with other omics approaches.	•Useful as starting point for the other emerging techniques, taken alone is not much informative.	(153, 154)
Single -omic	Large-scale studies which refer to the systematic identification and quantification of the overall components of a specific cell domain (such as transcriptome, proteome, metabolome, lipidome…).	•Quite a complete overview on a single aspect of a sample of cells. •Well-assessed and validated methods. •At the level of Transcriptome is feasible also in patients but expensive, while for proteomic, metabolomic and lipidomic is still far from patients.	•Single-omics data offer only a limited amount of information on biological mechanisms restricted to a single field.	(155–157)
Multi -omics	Combination and integration of several methods and data sets of different -omic groups during the biological analysis.	•A potent integrative approach, which provides a high amount of information, allowing to connect a genotype to a phenotype for a full cellular readout. •Depending on the type of analysis and data sets employed it can lead to the direct measure of causes and consequences of biological phenotypes.	•Highly expensive.	(158, 159)
Spatial biology	Combination of different techniques of sequencing and imaging (such as MERFISH/SeqFISH, CyCIF,IMC…) in order to examine the types of cells, their distribution throughout the tissue, the patterns of biomarker co-expression, and the organization and cross-talk in their microenvironment.	•Learn new biological insights by analyzing cells in their environment.	•Highly expensive. •Need of specialized facilities. •Complexity of data analysis.	(160–171)

### 7.1 Single-cell RNA sequencing

The important next step in data generation and subsequent analysis occurred with single-cell experiments, leading to the opportunity of analyzing the transcriptome at the single-cell level for millions of cells in a single study. Single-cell RNA sequencing enables scientists to characterize, discriminate, and identify each cell at the transcriptome level, leading to the finding of rare but functionally significant cell sub-populations (153).

Today, a growing number of modified and enhanced single-cell RNA sequencing technologies have been designed to bring important adjustments and improvements in sample collection, single-cell capture, barcoded reverse transcription, complementary DNA (cDNA) amplification, library preparation, sequencing, and refined bioinformatics analysis. Most importantly, the cost has been drastically decreased, while throughput and automation have both been greatly boosted (153).

In single-cell RNA sequencing, single cells are isolated from tissue samples, captured, and then combined with a bead inside a nanoscale droplet (each bead contains unique molecular identifiers). Barcoding, cDNA amplification, and the library preparation steps follow this stage. In order to present and categorize the landscape of gene expression in cells of a heterogeneous population, snapshot data from single-cell RNA sequencing can be examined (153, 154).

Single-cell sequencing technologies’ most recent technical and computational advancements have greatly expanded researchers’ toolkits for studying TME directly from patient tissues. BC is just one of the many tumor types for which single-cell RNA sequencing has been extensively employed to depict the intra-tumoral immune landscape (155).

For instance, despite the immune checkpoint blockade (ICB) therapy having produced impressive and long-lasting clinical responses in a limited number of cancer patients, its overall response rate has been low, and many patients with initial responses have refractory disease or have developed acquired resistance. The observed variability in ICB efficacy has been associated with a number of TME-related factors, through single-cell analysis, specifically with markers of the intra-tumoral T cell states, such as overall T cell infiltration, activation, and exhaustion. In fact, the enhancement of single-cell transcriptomic tools applied to TME studies improved our knowledge of tumor complexity, adaptability, and its intricate cross-interaction between various cell types within the TME (20, 155, 156).

With a similar approach, Gambardella et al. studied tumor heterogeneity and drug response, providing a transcriptional analysis of several BC lines. They demonstrated that the expression of clinically important markers could be detected through single-cell transcriptomics. Furthermore, they showed that different cells within the same BC cell line express heterogeneously relevant well-known BC receptors, including PR and HER2. Additionally, they observed dynamic plasticity as a consequence of drug responsiveness (157). In particular, they developed a bioinformatics tool that, starting from single-cell profiles, leads to drug response prediction at the single-cell level, firstly detecting expression-based biomarkers of drug sensitivity for several drugs, then correlating them with drug potency in different cellular lines. To experimentally validate their bioinformatic tool, they applied it to a BC cell line, the MDA-MB-361, identifying and sorting two cell subpopulations for HER2 receptor expression. Based on their computational prediction, they tested in both cell types representative drugs, obtaining results in line with the expected outcome (157).

Moreover, in line with these findings, the massive parallel sequencing and other omics technologies have demonstrated the level of heterogeneity in TNBCs, underling the potential impact of TME in therapeutic responses (152).

### 7.2 Multi-omics

Researchers can now study and define the TME at single-cell resolution thanks to the advent of multimodal omics technologies, which presents an unprecedented opportunity to comprehend the heterogeneous complexity of the TME (158). In fact, an efficient way to connect the patients’ genetic background with a condition or trait is through genome-wide association studies, which connect genotype to phenotype, and multi-omics provides a potent integrative approach, as it consists of the combination of data sets of different omic groups during the biological analysis. Indeed, multi-omics data must be integrated to increase the accuracy of predicting the biological relationship between genotype and phenotype because single-omics data only offer a limited amount of information on biological mechanisms (132, 172). Moreover, a good practice is to further integrate the obtained multi-omics data with online available raw and processed data in the same field in order to generate reproducible and reliable results through different open datasets (173) (**Figure 4**).

There is an emerging need to construct integrated multi-omics data databases, such as the Multi-Omics Breast Cancer Database (MOBCdb) proposed by Xie and colleagues (174). It is an available library that incorporates clinical, genomic, transcriptomic, epigenomic, and treatment response data of many BC subtypes. By using several search methods, users of MOBCdb can receive information on single nucleotide variation, gene expression, microRNA expression, DNA methylation, and particular pharmacological responses. With this online resource, users have access to integrated multi-omics data of various BC subtypes, allowing the identification of possible new biomarkers for personalized medicine (174). Another example comes from the work by Fan et al., which provides insights into the molecular pathways behind BC prognosis, building a dataset of gene-interaction networks in BC and describing genes linked to long-term BC survival (132). Indeed, emerging evidence attributes multi-omics data integration to a prognostic value; for instance, Nguyen and colleagues identified two therapeutically relevant molecular subgroups of BC with subgroup-specific characteristics employing multi-omics datasets. These approaches hold the promise for the creation of specific diagnostic tests and personalized medicine (175).

As TME components have been shown to play a crucial role in the occurrence, growth, and metastasis of BC, the development of single-cell omics largely addressed the limitations of purely biological assays and allowed us to comprehend the changes in cell populations, metabolic profiles, and immunological state of the TME throughout tumor progression. Now there is a better understanding of tumor complexity thanks to the ongoing development of integrated tools for single-cell omics that not only detect cell heterogeneity but also expand analysis for transcription-based cell cloning aberration (176), cell traceability (177), cell-to-cell interaction (178, 179), rare cell resolution (180), and disease process simulation (181). The recent single-cell omics results have mapped out breast TME with fairly high accuracy, sorting stromal cells and immune cells into functional populations and significantly employing the TME components for clinical diagnosis and targeted treatment intervention (182).

In particular, TNBC heterogeneity is characterized by genomic instability and elevated mutation rates, with an impact on immune surveillance (183). Therefore, to pinpoint the therapeutic approach for TNBC, specific driver genes, and pathways should be determined for better patient subtyping and target therapy (184). The most frequently altered genes, the genetic profile most likely contributing to the malignancies’ development, and the genes associated with metastatic TNBC can all be identified through recent advancements in whole genome sequencing. Emerging targeted therapies may enhance therapeutic effects by overcoming drug resistance and promoting patients’ survival (183, 184). For instance, Xiao et al. performed an extensive immunogenomic analysis to investigate the heterogeneity and prognostic importance of the TNBC microenvironment using a big original multi-omics dataset of TNBC. They also investigated TNBC’s potential immunological escape pathways. This trial is a step in the direction of individualized immunotherapy for TNBC patients, as TME phenotypes were classified in different clusters and validated with a significant prognostic efficacy (185).

Furthermore, Xie et al. focused their study on FOXO family genes and their correlation with TME in several cancers, including breast. As they examined the relationship between the FOXOs score and a variety of drugs, they showed that the FOXOs score might reflect patients’ responses to different therapies. They discovered that the majority of drugs’ IC50 values in pan-cancer, but particularly in BC, were negatively linked with the FOXOs score, corroborating the theory that a high FOXOs score would make a patient more susceptible to chemotherapy and targeted medications. Collectively, their data show that the FOXOs score has a substantial correlation with the TME and may be used as a biomarker to predict the effectiveness of various treatments, including immunotherapy (159).

### 7.3 Spatial biology and phenotyping

**Figure 5:**
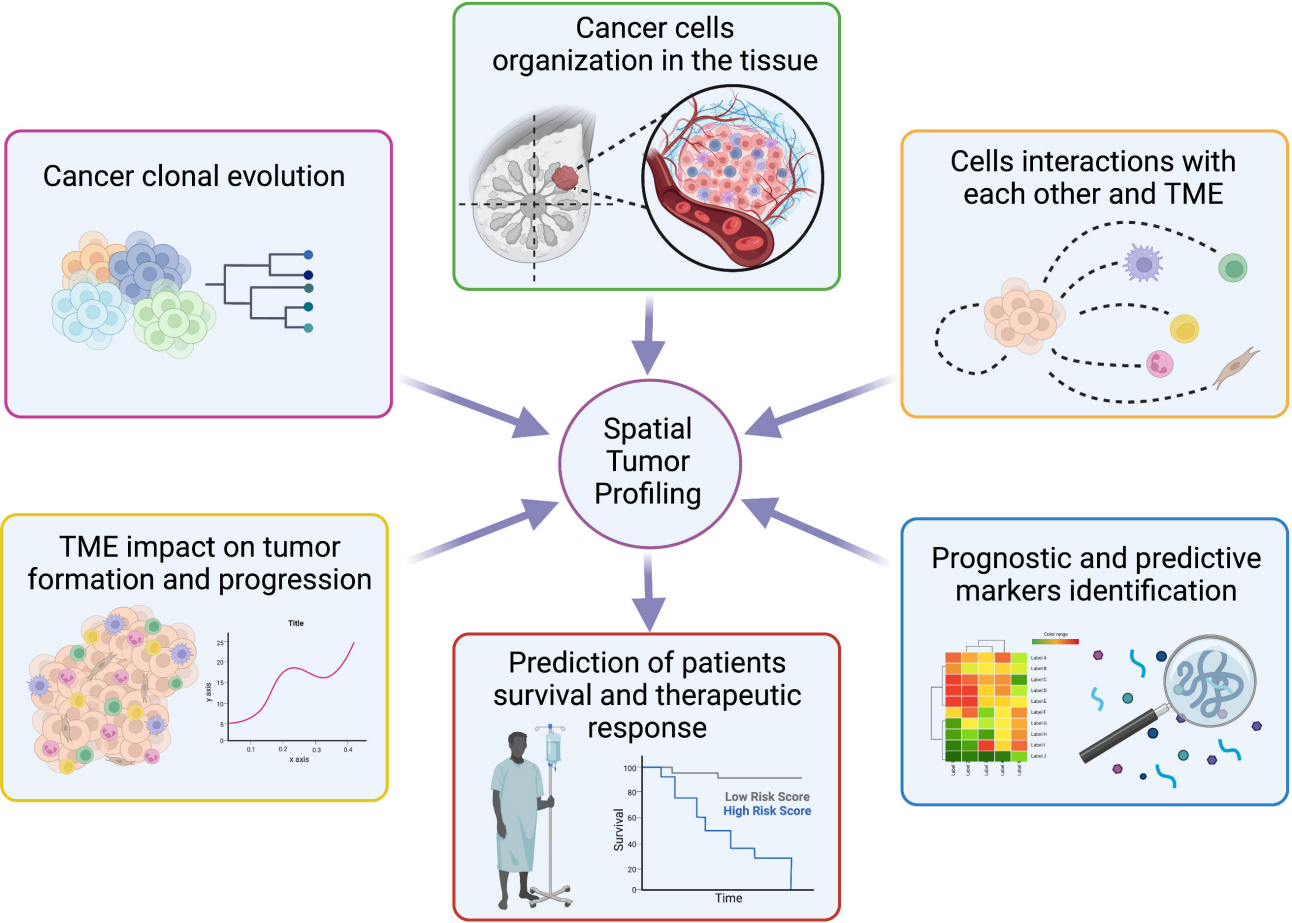
Overview of spatial tumor profiling applications. Schematic representation of the main opportunity offered by spatial techniques in the molecular biology field with possible applications to personalized medicine. Created with BioRender.com.

The study of a variety of cellular landscapes across different dimensions is known as spatial biology. Studies of spatial biology examine the types of cells, their distribution throughout the tissue, the patterns of biomarker co-expression, and the organization and interactions that compose the TME, allowing to learn new biological insights by analyzing cells in their environment (**Figure 5**) (160–162).

Methods for spatial molecular profiling have dramatically increased recently and differ in resolution, scale, and molecular multiplexing, with an improvement of spatial methods, in particular for transcriptomic, proteomic, and metabolomic (160). Different length scales are used by methods to capture a variety of data: from averages across cells spanning thousands of micrometers in spot-based procedures like spatial transcriptomics to single-molecule resolution in methods like MERFISH or SeqFISH. Consequently, a variety of questions can be addressed using the most suitable technique. The number of molecular features acquired varies between methods as well, ranging from tens in Fluorescence *In Situ* Hybridization (FISH), Cyclic Immunofluorescence (CyCIF), and Imaging Mass Cytometry (IMC) to hundreds or thousands in specialized probe-based spatial transcriptomics methods (MERFISH or SeqFISH), imaging mass spectrometry, and tens of thousands in spot-based spatial transcriptomics, including Slide-Seq, Visium and High-Definition Spatial Transcriptomics (HDST) (160, 161, 163). To detect and quantify biomarkers expression as well as to visualize how cells interact and organize throughout the entire tissue landscape, true spatial biology investigations exploit whole-slide imaging at single-cell resolution. This method is also known as spatial phenotyping.

An interesting use of this new technological branch is the one applied to the study of TME. Studies utilizing spatial multi-omics methods have demonstrated the complexity of the TME heterogeneity and showed that, in addition to the cellular composition, the relative localizations and interactions with different cell types in the TME significantly impact tumor formation. In fact, a better understanding of spatial interactions led to the redefinition of tumor subtypes and the shifting of research attention to tumor-immune interaction units, to the discovery of additional cell types, and of the changes in the TME compartment throughout cancer progression (160, 164). For example, in BC, the different subclones that contribute to the heterogeneity of the cancer mass were found to map in distinct regions (165, 166), with a specific architecture suggestive of a deep role of the TME, where CAFs show great heterogeneity and spatial separation (167). Liu et al. provide a novel insight into the cellular architecture of BC and potential therapeutic strategies, revealing differential association with patient survival and therapeutic response through single-cell and spatially resolved analysis (165). The analysis of two BC samples showed that malignant subclones map to regions of stromal cell enrichment, indicating that, even if only two BC were analyzed in detail, the diverse abundance of genetically and spatially distinct subclones is differently associated with patient survival and therapeutic response (165). This evidence suggests that it might be worth investigating how the heterogeneous architecture of cancer cells impact therapy response.

Moreover, the treatment of several cancers has been transformed by ICB. However, unfortunately, most patients only have minimal benefit from ICB, even after an initial response. Multi-omics TME assessment is indeed required for precision immune oncology in order to discover distinctive prognostic features and proactively personalize combinatorial treatments. Through accurate epitope colocalization, multiplexed single-cell spatially resolved tissue analysis enables the discernment of cellular functional states from their spatial organization, and emerging markers evaluated in multiplexed spatial protein analysis may help determine prognostic and predictive patterns in BC (168).

In particular, the work from Tietscher and colleagues suggests that single-cell data used for a comprehensive, spatially resolved, immune-focused analysis of TME could be useful for patient stratification to select them for ICB therapy. Indeed, they have defined two unique immunological microenvironments in breast tumors; each one may influence the response to immunotherapy, considering tumor antigen presentation, T cell phenotypes, cytotoxic potential, cellular interaction, and spatial organization. As the primary marker currently utilized to stratify patients for immune checkpoint treatment in BC is PD-L1, their findings imply that PD-1, CXCL13, and MHC-I, possibly in combination with previously identified T cell exhaustion markers like LAG-3 and TIM-329, are more effective at differentiating immunological TME that may show to be differentially receptive to this treatment. Therefore, these variables may be helpful in selecting individuals for prospective clinical trials of ICB, along with other patient stratification techniques (169).

Further work by Kulasinghe et al. provides new insights into the TNBC TME and its association with chemotherapeutic response and patient survival (170). In particular, spatial studies on TNBC samples revealed differentially expressed proteins and protein signatures within tumors and stroma compartments that associate with prognosis (overall survival) and treatment response. Following this approach, they were effectively able to stratify patients by their response to therapy (170).

Overall, recent evidence indicates that cancer study in a spatial context will improve the current knowledge of how the complex cross-talk between tumor and surrounding microenvironment results in the malignant subclones’ growth and progression, with an impact on survival and resistance to therapies (**Figure 5**) (171).

## 8 Mathematical modelling and artificial intelligence in unraveling BC complexity

### 8.1 Mathematical modelling

As well known, the origins of BC heterogeneity lie in both the stochastic nature of biological phenomena and their nonlinear dynamics. In these contexts, small changes in the complex interactions among different genetic, epigenetic, and environmental factors can have dramatically different effects on the evolution of the biological system. This strong dependence on probabilistic mechanisms and initial conditions makes it extremely difficult to fully understand the mechanisms and implications of heterogeneity in breast cancer.

To address this challenge, mathematical models have been developed and widely used to study and understand the complex processes underlying breast cancer heterogeneity. These models are based on mathematical equations, both deterministic or probabilistic, and simulations that allow researchers to investigate how different factors interact with one another and how they contribute to the development of the disease.

Examples of such models date back to at least a decade ago (48), with studies connecting different axes of phenotypic plasticity to explain the emergence of heterogeneity and drug resistance (186). These models have shown how genetic and epigenetic changes can lead to the development of different breast cancer subtypes, and how these subtypes can respond differently to different treatments.

Additionally, mathematical models have shown how a single axis of plasticity can give rise to extensive diversity upon mutations (187). This has important implications for the development of new treatments, as it suggests that targeting specific mutations may not be sufficient to treat breast cancer effectively. Instead, a more comprehensive understanding of the underlying mechanisms and interactions is needed to develop targeted and effective treatments.

In particular (48), and (187) highlight how the heterogeneity of breast cancer rests on a stochastic and combinatorial nature of the genetic and epigenetic elements that could interact together.

Finally, an additional dimension of non-linearity was highlighted in two different types of epithelial-mesenchymal transition (EMT) dynamics: one hysteretic and one non-hysteretic (188). In particular, specific gene patterns characterized by significant clinical prognosis value were highlighted in the EMT hysteretic dynamics.

These modeling efforts have been demonstrated to be useful for implementing therapeutic targets *in vivo*, allowing researchers refine their understanding of the mechanisms underlying breast cancer, to test the efficacy of new treatments and improve the effectiveness of therapies (189).

In summary, mathematical models have proven to be valuable tools for understanding the complexity of breast cancer heterogeneity while providing predictive tools on how biological systems may respond to specific perturbations.

Integration with increasingly precise and specific data, derived, for example, from scRNA_Seq techniques, will allow these models to help shed light on the mechanisms underlying the disease and develop more effective treatments for patients. The availability of increasingly rich and detailed databases has enabled the application of powerful and versatile Machine Learning/Artificial Intelligence systems to model and attempt to decipher additional levels of non-linear characters of this heterogeneity as discussed in the next paragraph.

### 8.2 Artificial intelligence in BC and TME

The use of Artificial Intelligence (AI) and Machine Learning (ML) is becoming widespread in the field of biology and life sciences (190). The existence of large databases, such as the one derived from the omics study in TME, necessitates the use of advanced tools and techniques. These new AI tools are rapidly becoming important for researchers (**Figure 4**) by leveraging the connection of large amounts of data and their elaboration, simplify the discovery of non-linear correlation in complex datasets. ML can be classified into three main categories: supervised learning, unsupervised learning, and reinforcement learning, where supervised learning involves learning from labelled data to make predictions, unsupervised learning involves finding patterns in unlabeled data, and reinforcement learning involve learning from interactions with an environment to maximize a reward signal (191).

Clustering is a popular omics analysis method that is used to find regularity and patterns in the data to help differentiate cancer molecular subtypes. One example of clustering is the assessment of immune cell infiltration levels using neural network techniques. This method can be used to classify patients based on the degree of immune cell infiltration in lung cancer (192).

Another important method is feature selection with Principal Component Analysis (PCA). This technique helps to reduce the dimensionality of the dataset, thus reducing the number of features. This can be done using different machine learning algorithms, such as Random Forest, that can be very useful in identifying genes that present a correlation with different types of cancer (193).

Feature transformation is another emerging approach that is still under development. This method aims to merge and modify existing features to create new ones. This can be helpful in merging different types of data, such as *in vitro* and clinical data. Feature transformation is an important approach to consider as it can uncover new insights and relationships in the data that are not immediately apparent.

A practical application of advanced machine learning to the study of TME in BC is DeepSpaCE (Deep learning model for Spatial gene Clusters and Expression), where advanced deep learning techniques have been applied in the context of spatial transcriptomics. In the paper (194), the authors applied a Convolutional Neural Network (CNN) to obtain a system able to reproduce with super-resolution the spatial gene expression from TME samples and then predict gene-expression levels in tissue sections within consecutive sections. This method enables users to derive hidden histological characters via spatial transcriptome and gene annotations, leading to accelerated biological discoveries without additional experiments. Indeed, they confirmed its performance using the spatial-transcriptome profiles and immunohistochemistry images of consecutive human breast cancer tissue sections. For example, the predicted expression patterns of SPARC, an invasion marker, highlighted a small tumor-invasion region that was difficult to identify using raw spatial transcriptome data alone because of a lack of measurements. They further developed semi-supervised DeepSpaCE using unlabeled histology images and increased the imputation accuracy of consecutive sections, enhancing applicability for a small sample size.

In particular, recent developments in ML algorithms have shown that deep learning (DL)-based models can recognize non-linear relationships in the data, along with linear relationships, from highly dimensional data derived from different -omics (195). In the context of omics and data analysis, ML, such as DL, can be used to identify patterns and correlations in the data that would be difficult or impossible to detect by humans (196, 197).

Finally, ML algorithms can also be applied to a well-curated scRNA-seq dataset of breast cancer patients. In this study (198), the authors developed an advanced ML model to identify cell lineage and subtypes and to automatically obtain the lowest unique molecular identifiers (UMI) threshold, reducing the time required for these analyses and simplifying the entire procedure.

## 9 Conclusions and perspectives

The complexity of BC, mainly due to the intra- and inter-tumor heterogeneity present in both primary tumors and metastatic lesions, represents a great obstacle in unraveling drug resistance mechanisms. In this Review, we have addressed the various determinants that contribute to BC heterogeneity, highlighting the primary role of the tumor microenvironment. We have deeply discussed the recent advances in uncovering BC heterogeneity, thanks to the ability to dissect the genetic diversity of cell subpopulations, the cancer cell plasticity, and the complexity of TME. We have also discussed the impact of tumor heterogeneity and TME on tumor progression and drug resistance, with the idea that the new molecular insights that are emerging need to be translated into improved therapeutics (**Figure 4**).

How to overcome cancer heterogeneity to improve cancer therapy remains a major challenge. Actually, mortality in BC is generally due to resistance to successfully treating metastatic disease. Metastases spawned via dissemination in different organs evolve in entities that are distinct from each other and from the primary tumor. Therefore, they need to be handled as independent tumors, with ongoing epigenetic evolution combined with the contribution of the specific TME in the metastatic site. Recent results also strongly put forward that dissimilar TME can affect the metastatic sites, leading to selection for survival and outgrowth of genetically different metastatic variants.

We describe the developments in single-cell RNA sequencing and in multi-omics technology on clinical samples, which are already providing insights into the phylogenetic correlation of primary tumors and metastases at the level of somatic tumor genetics, and that can reveal fundamental mechanisms of the metastatic process. However, deeper insights are needed to study variations that occur at the epigenetic level of matched primary and metastatic tumors of larger numbers of patient and experimental tissue samples. An emerging field to take into account is also the importance of the personal genetic landscape of each patient, which can strongly modify tumor and metastasis biology, their response to TME, and their drug responsiveness.

The *in vivo* preclinical models remain fundamental to study processes that cannot be readily inquired in humans and also to allow the study of the natural history of disease progression in the untreated state. However, since these studies are costly and labor-intensive, the bright use of mouse models should take into account the features that differ from human BC. Preclinical models, therefore, offer an important vehicle for generating and testing hypotheses that can then be validated across the broader human population. Where possible, models should include an intact immune system, the humanized mice being important platforms to overcome species-specific differences in the genetics and immune system between mice and humans. Moreover, attempts should be made to incorporate genetic heterogeneity in study designs by using several diverse models on different genetic backgrounds. On the other hand, *in vitro* organoid models derived from GEMMs or human clinical material could offer new perspectives in terms of rapid functional screening of genes and pathways that can influence tumor progression and of availability of material to analyze epigenomics and chromatin landscape evolution that is currently difficult to do using only human biopsies.

In conclusion, overcoming cancer heterogeneity to date remains a difficult task. In terms of TME, a better understanding of its complex organization, spatial heterogeneity, and changes in metastatic progression under the pressure of therapy is crucial for patient survival. The available targets are few and this field still needs further research. In the meantime, it’s worth mentioning that using a combination of drugs can increase therapy response, likely due to the synergistic effect of the drugs in selectively killing cancer cells and creating a more restrictive environment. In addition, high-resolution sequencing techniques prior to therapy and longitudinal sampling can provide a good source of information to delineate the optimal therapeutic strategy.

As shown by Navarro Ocon et al., new nanomedicine-based therapies have been proposed to alleviate immunosuppression in tumors and reduce the emergence of tumor heterogeneity in BC patients. Indeed, nanomedicine can improve the delivery, retention, and release of immunostimulatory agents in targeted cells and tumor tissues in numerous malignancies, including breast cancer (199). Moreover, the goal of finding new ways to revert a hostile TME by immune-activating cytokines is frequently hampered by the severe toxicity associated with their systemic administration. Very recent works in mouse models of glioblastoma (GBM) (200), melanoma, and mammary tumors (201) demonstrated a TME reprogramming toward anti-tumor activity upon targeted delivery of IL-12 via different approaches. In particular, Birocchi et al. describe a lentiviral vector-based platform that can engineer hematopoietic stem cells *ex vivo* with the aim of releasing, via their tumor-infiltrating monocyte/macrophage progeny, Interferon alpha (IFN-α) or IL-12 at the tumor site with spatial and temporal selectivity. In a preclinical syngeneic GBM mouse model, the inducible release of IFN-α within the TME achieved strong tumor inhibition up to eradication and outperformed systemic treatment with the recombinant protein in terms of efficacy, tolerability, and specificity. Single-cell RNA sequencing of the tumor immune infiltrates revealed reprogramming of the immune microenvironment toward a proinflammatory and antitumoral state, demonstrating a potential therapeutic approach for GBM (200) and paving the way to treat with locally delivered IL-12 other solid tumors, including melanoma and BC.

## Author contributions

VS, GC, LA, DN, AP, and PA designed the structure of the review and analyzed the state of the art in the field UA, DT, ET, and PD supervised data collection. All authors wrote the review. All authors contributed to the article and approved the submitted version.
